# Long-Read Isoform Sequencing Reveals a Hidden Complexity of the Transcriptional Landscape of Herpes Simplex Virus Type 1

**DOI:** 10.3389/fmicb.2017.01079

**Published:** 2017-06-20

**Authors:** Dóra Tombácz, Zsolt Csabai, Attila Szűcs, Zsolt Balázs, Norbert Moldován, Donald Sharon, Michael Snyder, Zsolt Boldogkői

**Affiliations:** ^1^Department of Medical Biology, Faculty of Medicine, University of SzegedSzeged, Hungary; ^2^Department of Genetics, School of Medicine, Stanford UniversityStanford, CA, United States

**Keywords:** human herpesvirus 1, herpes simplex virus type 1, transcriptome, transcriptional interference, full-length sequencing, long-read sequencing, Pacific biosciences, transcriptional overlap

## Abstract

In this study, we used the amplified isoform sequencing technique from Pacific Biosciences to characterize the poly(A)^+^ fraction of the lytic transcriptome of the herpes simplex virus type 1 (HSV-1). Our analysis detected 34 formerly unidentified protein-coding genes, 10 non-coding RNAs, as well as 17 polycistronic and complex transcripts. This work also led us to identify many transcript isoforms, including 13 splice and 68 transcript end variants, as well as several transcript overlaps. Additionally, we determined previously unascertained transcriptional start and polyadenylation sites. We analyzed the transcriptional activity from the complementary DNA strand in five convergent HSV gene pairs with quantitative RT-PCR and detected antisense RNAs in each gene. This part of the study revealed an inverse correlation between the expressions of convergent partners. Our work adds new insights for understanding the complexity of the pervasive transcriptional overlaps by suggesting that there is a crosstalk between adjacent and distal genes through interaction between their transcription apparatuses. We also identified transcripts overlapping the HSV replication origins, which may indicate an interplay between the transcription and replication machineries. The relative abundance of HSV-1 transcripts has also been established by using a novel method based on the calculation of sequencing reads for the analysis.

## Introduction

Herpes simplex virus type 1 (HSV-1) is a human pathogenic alphaherpesvirus from the *Herpesviridae* family. Herpes is a lifelong infection, which often has mild or no symptoms. The most common symptoms of viral infection are cold sores. HSV-1 can cause acute encephalitis in immunocompromised patients. According to WHO's first global estimates, worldwide more than 3.7 billion people under the age of fifty are infected with HSV-1 (Looker et al., 2015). The HSV-1 genome is composed of a unique long (UL) and a unique short (US) region, both being bracketed by inverted repeats (IRLs and IRSs, respectively). According to earlier annotations, the HSV-1 DNA contains 89 protein-coding, 10 long non-coding (lnc)RNA genes (Rajčáni et al., 2004; McGeoch et al., 2006; Macdonald et al., 2012; Lim, 2013; Hu et al., 2016) and several micro RNAs (Du et al., 2015).

Herpesvirus genes are expressed in a coordinated temporal cascade and grouped into three kinetic classes, immediate-early (IE), early (E), and late (L) (Harkness et al., 2014). The IE proteins are required for the transcription of both E and L genes. The E genes typically encode proteins that play a role in DNA replication, while the L genes specify the structural components of the virus. Kinetic analysis of the HSV transcriptome faces a significant challenge due to the overlapping nature of the viral genes. The typical architecture of polycistronic units is characterized by varying transcription start sites (TSSs) that are caused by the control of distinct promoters, and shared transcription end sites (TESs). As an example, the following transcripts are produced from a tetracistronic unit: 1-2-3-4, 2-3-4, 3-4, and 4, where “1” represents the most upstream gene, while “4” is the most downstream gene within the given unit. In a recent study on the transcriptome of another alphaherpesvirus, the pseudorabies virus (PRV), we demonstrated that additional gene combinations are also produced mostly in the form of low-abundance RNA molecules (Tombácz et al., 2016). The downstream genes on the polycistronic transcription units are generally thought to be untranslated because the eukaryotic RNAs—with some exceptions (Craigen and Caskey, 1987)—use cap-dependent ribosome-binding sites at their 5′-ends (Merrick, 2004).

It has been demonstrated that a large part of the mammalian genome is transcribed, producing a large variety of non-coding (nc)RNA molecules (Bertone et al., 2004). There is a current debate on whether the genome-wide expression of ncRNAs merely represents transcriptional noise, or whether these molecules might have yet undisclosed functions (Mattick, 2004). The most abundant class of the non-coding transcripts are the lncRNAs (Mattick and Makunin, 2006), which are defined as RNA molecules that exceed 200 nucleotides. A large number of lncRNAs have recently been described in mice and humans (Wilusz et al., 2009). Several genomic regions containing protein-coding genes also encode antisense lncRNAs from the complementary DNA strand. The HSV LAT was described as the first viral lncRNA (Stroop et al., 1984). In a recent study, we have shown that both DNA strands along the entire PRV genome are transcriptionally active (Tombácz et al., 2016). Some evidence suggests that the extensive transcriptional read-throughs produce very long RNA molecules including some containing oppositely oriented ORFs, which have been classified as complex transcripts (Tombácz et al., 2016).

Alternative splicing expands the coding capacity of metazoan genomes by producing multiple messages from a single gene. The splice variants can have similar or antagonistic functions (Boise et al., 1993). Previously, only five HSV-1 genes had been reported to produce spliced transcripts (Rajčáni et al., 2004; Sedlackova et al., 2008).

Massively parallel DNA sequencing technologies have become useful tools for the analysis of the transcriptomes. However, the conventionally applied short-read platforms cannot reliably distinguish between transcript isoforms and overlapping RNA molecules. Currently, the most commonly used long-read technology is the single-molecule real-time (SMRT) sequencing platform developed by Pacific Biosciences (PacBio). This approach is capable of reading cDNAs generated from full-length transcripts in a single sequencing run permitting mapping of the transcript ends with base-pair precision.

Various methods have already been used for the analysis of the herpesvirus transcriptome including microarrays (Stingley et al., 2000), Illumina sequencing (Harkness et al., 2014; Oláh et al., 2015), multi-time-point real-time reverse transcription PCR (qRT-PCR) analysis (Tombácz et al., 2009), and PacBio SMRT sequencing (O'Grady et al., 2016; Tombácz et al., 2016, 2017). Next-generation sequencing platforms have only been used for analyzing the transcriptional activity along the viral genome (Harkness et al., 2014). The PacBio long-read sequencing approach used for transcriptome profiling of PRV has proven to be the most effective platform for the identification of polycistronic RNA molecules, complex transcripts, transcript isoforms, and transcript overlaps. Our multiplatform-based approach has revealed a previously unexplored complexity of the PRV transcriptome (Tombácz et al., 2016).

In this study, we report the application of PacBio long-read sequencing technology for the characterization of the global lytic transcriptome of HSV-1. We used an amplified isoform sequencing (Iso-Seq) protocol that was based on PCR amplification of the cDNAs prior to sequencing. Besides the identification of novel transcripts and transcript isoforms, our intention was to map the TSSs and TESs of the viral RNAs with base pair precision. Long-read sequencing has proved to be efficient in the identification of overlapping and alternatively processed transcripts, as well as other long RNA sequences. Additionally, this method is particularly suitable for the sequencing of small genomes, GC-rich DNAs (HSV-1 has a 68% GC content) and with many repetitive sequences (Tombácz et al., 2014; Scott and Ely, 2015). The PacBio sequencing technique has an additional advantage over the short-read platforms in that if any random errors occurs in the raw reads, they are easily corrected thanks to the exceptionally high consensus accuracy of this platform (Miyamoto et al., 2014). In this report, we reevaluated the currently available datasets concerning the polyadenylated fraction of the HSV-1 transcripts generated in productively infected cells.

## Materials and methods

### Cells and viral infection

An immortalized kidney epithelial cell line (Vero) isolated from African green monkey was used for the propagation of HSV-1 strain KOS. Vero cells were cultivated in Dulbecco's modified Eagle medium supplemented with 10% fetal bovine serum (Gibco Invitrogen) and 100 μl penicillin-streptomycin 10K/10K mixture (Lonza)/ml and 5% CO_2_ at 37°C. For the preparation of viral stocks, rapidly-growing semi-confluent Vero cells were infected at a multiplicity of infection (MOI) of 1 plaque-forming unit (pfu)/cell, followed by incubation until a complete cytopathic effect was observed. The infected cells were then frozen and thawed three times, followed by centrifugation at 10,000 × g for 15 min. The titer of the virus stock was determined in Vero cells. For the sequencing analysis cells were infected with MOI = 1, incubated for 1 h, followed by removal of the virus suspension and washing with PBS. This was followed by the addition of fresh culture medium to the cells, which were incubated for 1, 2, 4, 6, 8, or 12 h. Samples taken from each experiment were then mixed for the sequencing analysis.

### Pacbio RS II sequencing—the amplified Iso-Seq protocol

#### Synthesis of cDNAs and preparation of SMRTbell template

Polyadenylated RNAs were isolated from total RNA by using the Oligotex mRNA Mini Kit (Qiagen) according to the manufacturer's instructions. The cDNA libraries were prepared using anchored oligo-dT primers for the reverse transcription. The cDNA production and the SMRTbell library preparation were performed with the Iso-Seq protocol of PacBio sequencing using the Clontech SMARTer PCR cDNA Synthesis Kit. We carried out either no Size Selection for the analysis of short viral transcripts, or performed SageELF™ and BluePippin™ Size-Selection Systems (Sage Science) for the isolation of long RNA molecules. The SMARTer PCR cDNA Synthesis Kit (Clontech) was used for the generation of the first-strand cDNAs. Subsequently, the single-stranded cDNAs were PCR-amplified using KAPA HiFi Enzyme (Kapa Biosystems) following PacBio's recommendations, which were as follows: initial denaturation was carried at −95°C for 2 min, followed by 16 cycles (the optimal cycle was determined in the optimization step) at −98°C for 20 s (denaturation), −65°C for 15 s (annealing) −74°C for 4 min (extension). The final extension was carried out at −72°C for 5 min. From the non-size selected samples 500 ng of each cDNA sample was applied for the SMRTbell template preparation, using the PacBio DNA Template Prep Kit 2.0. Subsequently, PCR products were pooled then size-selected with the SageELF™ System according to the PacBio's protocol. Size-selected samples were amplified with KAPA enzyme using the conditions as above. The fraction of cDNAs with a size over 5 kb was run on BluePippin™ System to remove the short SMRTbell templates.

#### PacBio sequencing

The purified SMRTbell templates were bound to v2 sequencing primers and polymerases by using the DNA/Polymerase Binding Kit P6 (P/N 100-356-300). The conditions for annealing of the primers and binding of polymerase were determined using the PacBio's Binding Calculator in RS Remote. The polymerase-template complexes were bound to magbeads using the PacBio MagBead Binding Kit. The samples were analyzed on an Agilent 2100 bioanalyzer. Sequencing was carried out by using the PacBio RS II sequencer with DNA Sequencing Reagent 4.0 (P/N 100-356-200). The length of each movie was 360 min. We applied the PacBio Iso-Seq protocol (SMRT Analysis version v2.3.0.; Chaisson and Tesler, 2012) for the analysis of the HSV-1 transcriptome.

### PCR analysis

The novel HSV transcripts identified by PacBio sequencing were validated by PCR analysis. SuperScript III reverse transcriptase (Life Technologies) was used to convert the RNA samples to single-stranded cDNAs according to the manufacturer's instructions. The cDNAs were amplified with the Veriti Thermal Cycler (Applied Biosystems), using AccuPrime™ GC-Rich DNA Polymerase (Invitrogen). The PCR reactions ran for 3 min at 95°C (denaturation), followed by 30 cycles at 92°C for 30 s, at 60°C for 30 s (annealing), and at 72°C for 10 s (extension). Finally, a 10 min elongation step ran at 72°C. The primers used in this study are listed in Table S1.

### Strand-specific real-time RT-PCR

The relative amounts of transcripts were calculated by real-time reverse transcription (RT)-PCR. Three parallel RT reactions were carried out using 70 ng of total RNA as template, Superscript III enzyme (Life Technologies) and anchored oligo(dT) primers. In order to control for potential DNA contamination, No RT reactions were carried out for each sample by omitting the RT enzyme. The PCR reactions were carried out in a volume of 20 μl with Absolute QPCR SYBR Green Mix (Thermo Scientific) containing 7 μl of cDNA solution diluted 10-fold, 1.5 μl of forward and 1.5 μl of reverse primers (10 μM each). The normalized relative expression ratios (R_x_) were calculated by the following formula:

$R_{x}=\frac{1}{E_{sample}{}^{Ct_{sample}}/E_{total}{}^{Ct_{total}}}$, where *E* is the amplification efficiency, *Ct* is the threshold cycle number. The mean expression value of all examined transcripts (*total*) in a given sample was used as a normalization factor for the transcripts (*sample*) to obtain *R*_*x*_ values. This method is similar to that used by Mestdagh et al. (2009). However, instead of using the *Ct* values alone, we used *E*^*Ct*^ for the calculation of the expression values. The Comparative Quantitation module of the Rotor-Gene Q software v2.3.1 (Qiagen) was used for the determination of *Ct* values. The efficiency of the reaction and the thresholds were automatically calculated by the software.

### Transcript quantitation using PacBio sequencing reads

The following criteria were set to count the number of reads aligning to each transcript isoform: Reads of Inserts (ROIs) containing poly(A) tails were assigned to a transcript, if the poly(A) site (PAS) of the read was no longer than 10 nts from the PAS of the transcript (in either direction), and if the 5′ end of the read was no longer than 10 nts upstream or 10 nts downstream from the TSS of the transcript isoform. Reads with long trimmed ends (>100 nts), or with many deletions, insertions or mismatches (>5% of the alignment) were discarded. Only the reads that contained a single poly(A) tail were counted. A set of DNA ladders was sequenced to determine the read length preference of the sequencing method. Equimolar molecular-weight size markers (Lambda DNA StyI, Lambda DNA PstI and phiX174 DNA HaeIII, resulting in 50 discrete fragments, between 15 and 19.329 nts) were sequenced along with the viral sequences. The number of reads for each fragment length was determined and their distributions were used to normalize read counts of the HSV-1 transcripts. When assigning reads to transcript isoforms, each read was weighted by the probability of sequencing a read of that given length.

### Prediction of *cis*-regulatory sequences of the HSV genes

The poly(A) signals were predicted by Dragon PolyA Spotter (DPS) (Kalkatawi et al., 2012) using Artificial Neural Network classifier. We determined the common transcription factor binding sites (−100 nts from transcription start) for common binding sites including TATA-box, GC-Box etc., using JASPAR POLII database (Mathelier et al., 2016) and FIMO (Find Individual Motif Occurrences) software (Grant et al., 2011). The threshold was set to *p* < 0.001. The best hits are linked to the genes and are included in Tables S2, S3.

## Results

### Analysis of the HSV transcriptome with PacBio isoform sequencing

The annotation of the herpesvirus genome has traditionally been carried out *in silico* by detection of ORFs and other cis-motifs. Many HSV-1 transcripts have been identified by Northern-blot analysis (Costa et al., 1984; Sedlackova et al., 2008), while the 5′ and 3′ termini of the transcripts have been determined by S1 nuclease mapping (McKnight, 1980; Rixon and Clements, 1982) or by primer extension (Perng et al., 2002; Naito et al., 2005) techniques. For the investigation of the HSV-1 lytic transcriptome, we employed a PacBio polyadenylation sequencing approach based on Iso-Seq template preparation protocol utilizing the switching mechanism at the 5′ end of the RNA template, which produces full-length cDNAs (Zhu et al., 2001). We mapped the ROIs for both the HSV-1 (X14112) and the host genome (NC_023642.1) using the GMAP (Wu and Watanabe, 2005) alignment tool with default parameters. Altogether, 50,166 ROIs were mapped for the viral genome with a mean length of 1,461 nts, representing 7.32 million bases; and 35,089 ROIs were mapped for the host genome with mean length of 1,262 nts, representing 4.43 million nucleotides. Size-selection was applied for the detection of transcripts exceeding 5 kbps. The criteria for accepting sequencing reads as existing transcripts were the presence of at least two independent ROIs which contained the same TSSs and TESs. Likewise, a novel splice site was accepted if the same site occurred in at least two independent ROIs. We ran 17 parallel sequencing reactions for providing independent sequencing reads. Additionally, in many cases, the same TSS, TES, or splice junction were also found in different transcripts detected within the same sequencing reaction, which further enhanced the number of independent ROIs. In most cases, even in low-abundance transcripts, far more than two independent ROIs contained the same transcript ends or splice junctions. Besides the poly(A) tails, a few sequencing reads were also produced from A-rich regions of the transcripts; these non-specific products were discarded from further analysis. A few ROIs possessed both poly(A) and poly(T) tails. This phenomenon was earlier described in human (Sharon et al., 2013) and PRV (Tombácz et al., 2016) transcriptomes that were sequenced using the PacBio technique. Altogether, we could predict TATA boxes for 77 HSV-1 transcripts (Table S2). The promoter regions of the other genes lack typical TATA boxes, but many of them contain GC- and/or CAAT boxes (Table S2).

### Novel putative protein-coding genes

**Figure 1:**
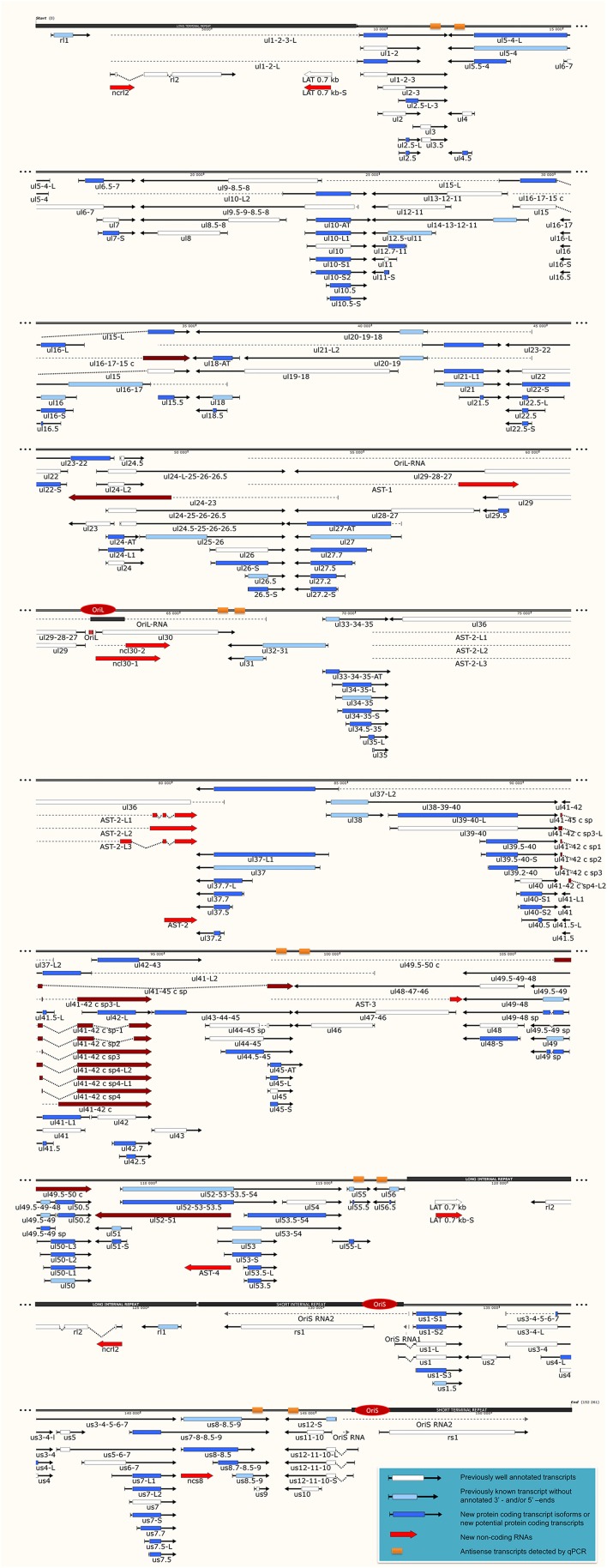
The transcriptome of the herpes simplex virus. The current version of the HSV-1 genome is composed of 115 protein-coding genes and 19 putative non-coding RNAs. The coding transcripts identified earlier are depicted as arrow-rectangles with white boxes (indicating the ORFs), the previously known transcripts (without annotated TSS and TES positions) are illustrated as light blue arrow-rectangles. The novel potential protein coding genes are labeled by dark blue rectangular arrows. The already identified lncRNAs are represented as dark-gray arrow lines, while the novel putative lncRNAs are depicted as red arrow lines. The novel polycistronic transcripts are indicated with dark blue rectangular arrows. The dark-red rectangular arrows show complex transcripts. The long black boxes represent the repeat regions of the HSV genome; red ovals represent the three replication origins. Abbreviations within the name of the transcripts: S, short TSS variant; L, long TSS variant; AT, alternative TES variant.

Our investigations revealed 34 novel putative protein-coding genes (Figure 1, Table 1, Table S4), which is a fairly large number in a well-characterized viral genome. Each new gene is embedded into already annotated protein-coding genes. The mRNAs from these 5′ truncated genes are generated by alternative transcription initiation from an intragenic promoter within the larger host gene. Embedded genes are well-known in the herpesviruses, and their number is increasing; for example, the detection of the novel *ul36.5* gene by PacBio sequencing has recently been described (Tombácz et al., 2016). The first in-frame AUG triplets are supposed to correspond to the first amino acids of the putative protein molecules. With the exception of seven genes (*ul5.5; ul15.5; ul21.5; ul24.5; ul27.2; ul41.5;* and *ul53.5*), the remaining novel genes were expressed at markedly lower levels than the host genes.

**Table 1:** Novel putative HSV genes.

**New genes**	**TSS**	**TES**
ul2.5	10,323	10,965
ul4.5	12,412	11,734
ul5.5	13,518	11,732
ul6.5	16,433	18,060
ul10.5	23,491	24,665
ul12.7	25,781	24,782
ul15.5	33,929	34,844
ul16.5	31,158	30,147
ul18.5	35,898	35,004
ul21.5	42,503	43,708
ul22.5	44,955	43,821
ul27.2	54,478	53,040
ul27.5	54,757	53,037
ul27.7	55,236	53,037
ul29.5	59,147	58,397
ul34.5	69,732	70,957
ul37.2	81,487	80,693
ul37.5	81,720	80,693
ul37.7	82,047	80,693
ul39.5	88,773	91,001
ul39.2	89,001	91,001
ul40.5	89,964	91,001
ul41.5	91,878	91,105
ul42.7	93,528	94,653
ul42.5	93,733	94,654
ul44.5	96,620	98,691
ul50.5	107,154	108,173
ul50.2	107,178	108,173
ul53.5	112,508	113,461
ul55.5	115,530	116,122
ul56.5	117,083	116,182
us7.7	139,907	141,033
us7.5	140,242	141,035
us8.7	142,071	143,687

*All of the identified candidate genes are located within the coding region of an already annotated HSV-1 gene. This table shows the list of the putative genes and the positions of their transcriptional start (TSS) and end (TES) sites*.

### Novel non-coding transcripts

**Figure 2:**
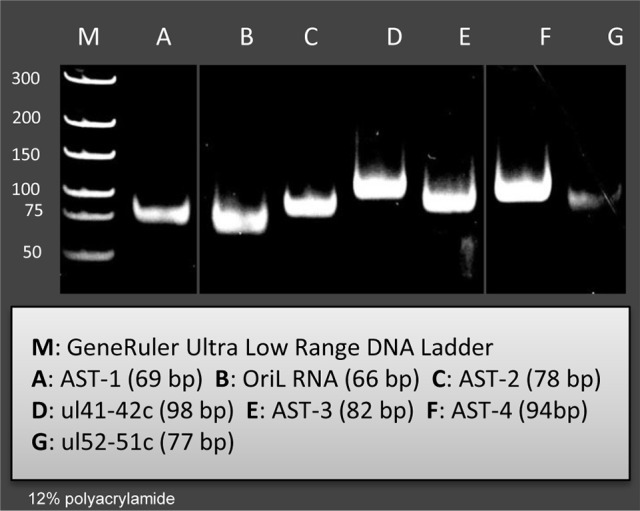
Validation and quantifying of antisense transcripts. Our PacBio analyses identified ten novel ncRNAs. We could confirm the expression of those transcripts with PCR analyses which were not embedded or overlapped with other transcripts. The following novel ncRNAs were detected by PCR (the sizes of PCR amplicons are in brackets): AST-1 (69 nts), (B2) OriL RNA (66 nts), AST-2 (78 nts), ul41-42c (98 nts), AST-3 (82 nts), AST-4 (94 nts), and ul51-52 c (77 nts).

In this part of our study, we identified 10 novel ncRNAs, including a LAT variant, four antisense (as) and four 3′ truncated transcripts, as well as a putative RNA molecule overlapping the replication origin (Ori-L) of the virus (Figure 1, Table 2). The novel 0.7 kb LAT-S transcript is a TSS isoform of the 0.7-kb LAT (Zhu et al., 1999). We detected polyadenylated asRNAs (termed antisense transcripts, AST) transcribed from the complementary DNA strands (the predicted TATA boxes are presented in Table S2). The existence of ASTs was verified by strand-specific PCR (Figure 2). We also detected lncRNAs, which were produced from protein-coding genes, but their transcriptions were prematurely terminated, and as a result lack stop codons. The potential function of these 3′ truncated RNA molecules remains obscure. The homolog of NCS8 transcript has been described in PRV (Tombácz et al., 2016). Furthermore, we identified a putative Ori-L-overlapping ncRNA with uncertain orientation, TSS and TES. We cannot exclude the possibility that it is not an individual RNA molecule but is rather the upstream region of a putative longer TSS isoform of the UL30 transcript. We did not detect HSV ncRNAs homologous to the PRV CTO-S transcript located in the close vicinity of the Ori-L of this virus (Tombácz et al., 2015). The probable reason for this is that the Ori-L is situated at different genomic loci in the two viruses. We could however detect the Ori-S-overlapping RNA described by Voss and Roizman (1988).

**Table 2:** Novel non-coding RNAs.

**Transcripts**	**TSS**	**TES**
AST 1	57,711?	59,429
AST 2	79,792	80,725
AST 3	103,150?	103,512
AST 4	112,131	110,816
ncl30-2	63,467?	64,730
ncl30-1	62,605	64,458
ncrl2	124,254	123,543
ncs8	141,170	142,087
OriL RNA	62,455?	63,439?

*We identified a novel LAT transcript, four antisense RNAs, four 3′ truncated transcripts, and a putative OriL RNA molecule. In this table, the transcripts are listed with their start (TSS) and end (TES) positions. The “?” indicates that we were unable to determine the exact 5′ ends of the given transcripts*.

### Determination of the 5′ and 3′ termini of the HSV transcripts

In this report, we precisely mapped the 5′ and 3′ ends of the HSV transcripts (Table S3) and re-annotated those that had not been well-established earlier. Our amplified Iso-Seq technique is capable of identifying the full-length transcripts without any loss at the terminal sequences. We found that most of the TSSs were located between 28 and 33 nts downstream from the TATA box. Altogether, we were able to identify 46 novel TSSs and 6 TESs in already described transcripts, as well as 16 TSSs in the novel transcripts.

### Transcription start site isoforms

PacBio cDNA sequencing uncovered 42 protein-coding and non-coding transcripts with alternative TSSs (Figure 1, Table 3). We could detect putative TATA boxes for six TSS variants; however, TATA-less genes have been reported to be common in eukaryotic organisms (Yang et al., 2007). Similar to PRV, we detected multiple TSSs for the HSV UL10 transcripts, which indicate that the complex regulation of this gene is conserved among herpesviruses. We found that the *ul22* and the *us7* gene contain two active TATA sequences, and seven genes (*ul2*; *ul6.5*; *ul12*; *ul22*; *ul51*; *us1*; *us7*) have at least two potential TATA boxes. We identified two transcripts that overlap the Ori-Ss of HSV (Figure 1) at their 5′-UTRs; both are long TSS isoforms of *us1* and *us12* genes. In contrast to the PRV *us1* gene that is located in the IRS, in the HSV only the promoter and a short 5′-UTR of this gene map to the repeat region. The Ori-S-overlapping US1 transcript is homologous to the PRV PTO-US1. Our analysis has revealed extensive minor variations in ~68% of the transcripts. These RNA molecules are controlled by the same promoter and vary in length from 1 to 10 nts at their 5′-ends (Table 3).

**Table 3:** Novel TSS isoforms.

**Transcripts**	**TSS**	**TES**
0.7 kb LAT—S	8,393/117,978	7,644/118,728
Ul1-2-L	9,149?	10,965
Ul1-2-3-L	9,201?	11,737
Ul2.5-L	10,323	10,965
Ul5-4-L	15,620	11,741
Ul7-S	16,992	18,060
Ul10-L2	22,249?	24,666
Ul10-L1	22,883	24,666
Ul10-S2	23,062	24,665
Ul10-S1	23,029	24,666
Ul10.5-S	23,518	24,665
Ul11-S	25,280	24,782
Ul15-L	28,415?	34,840
Ul16-S	31,511	30,148
Ul16-L	31,832	30,147
Ul21-L2	41,452?	43,712
Ul21-L1	41,795	43,708
Ul22.5-S	44,571	43,818
Ul22.5-L	45,494	43,821
Ul22-S	46,554	43,823
Ul24-L1	47,468	48,767
Ul26-S	50,659	52,786
Ul26.5-S	51,715	52,786
Ul27.2-S	54,275	53,037
Ul34-35-L	69,335	70,959
Ul34-35-S	69,498	70,962
Ul35-L	70,136	70,959
AST 2-L1	79,449?	80,425
AST 2-L2	79,374?	80,425
AST 2-L3	78,531?	80,425
Ul37.7-L	82,293	80,693
Ul37-L1	84,467	80,692
Ul37-L2	84,757?	80,692
Ul39-40-L	86,168?	91,001
Ul39.5-40-S	88,808	91,001
Ul40-S1	89,816	91,001
Ul40-S2	89,856	91,001
Ul41.5-L	91,878	91,105
Ul41-L1	92,882	91,096
Ul41-L2	93,244?	91,096
Ul41-42 c p-3-L	91,013	94,657
Ul41-42 c p-4-L2	91,299	94,656
Ul41-42 c sp-4-L1	91,457	94,656
Ul42-L	92,666	94,652
Ul45-L	97,909	98,691
Ul45-S	98,029	98,691
Ul48-S	105,150	103,515
Ul50-L3	106,610?	108,173
Ul50-L2	106,704	108,173
Ul50-L1	106,800	108,173
Ul51-S	109,167	108,262
Ul53-S	111,943	113,461
Ul53.5-L	112,489	113,461
Ul55-L	115,027	116,122
Us1-S1	132,540	133,960
Us1-S2	132,551	133,960
Us1-S3	132,622	133,960
Us4-L	136,184	137,525
Us7-L1	139,190	141,035
Us7-L2	139,554	141,037
Us7-S	139,720	141,037
Us7.5-L	140,128	141,037

*In this part of our work, we detected novel TSS isoforms of both previously known and newly identified transcripts. This table shows the TSS and TES positions of the transcripts. The “?” indicates that we could not determine the exact 5′ end of the given transcripts*.

### Transcription end site isoforms

It has earlier been shown that many eukaryotic genes produce TES isoforms using alternative PA signals (Edwalds-Gilbert et al., 1997; Shen et al., 2008; Proudfoot, 2011). We detected six HSV transcripts, which each had two TES isoforms (Figure 1, Table 4). Except for three transcripts, we found that the rest of the abundant RNA molecules containing the same PA signal exhibited considerable polymorphism at their 3′-ends (Table 4).

**Table 4:** Novel TES isoforms.

**Transcripts**	**TSS**	**TES**
Ul10-AT	22,943	24,776
Ul18-AT	36,249	34,913
Ul24-AT	47,673	48,616
Ul27-AT	56,080	52,796
Ul33-34-35-AT	69,065	71,008
Ul45-AT	97,953	98,965

*We determined novel alternative polyadenylation sites for already identified protein coding transcripts. This table lists the newly identified transcripts with their TSS and TES positions*.

### Splice isoforms

**Figure 3:**
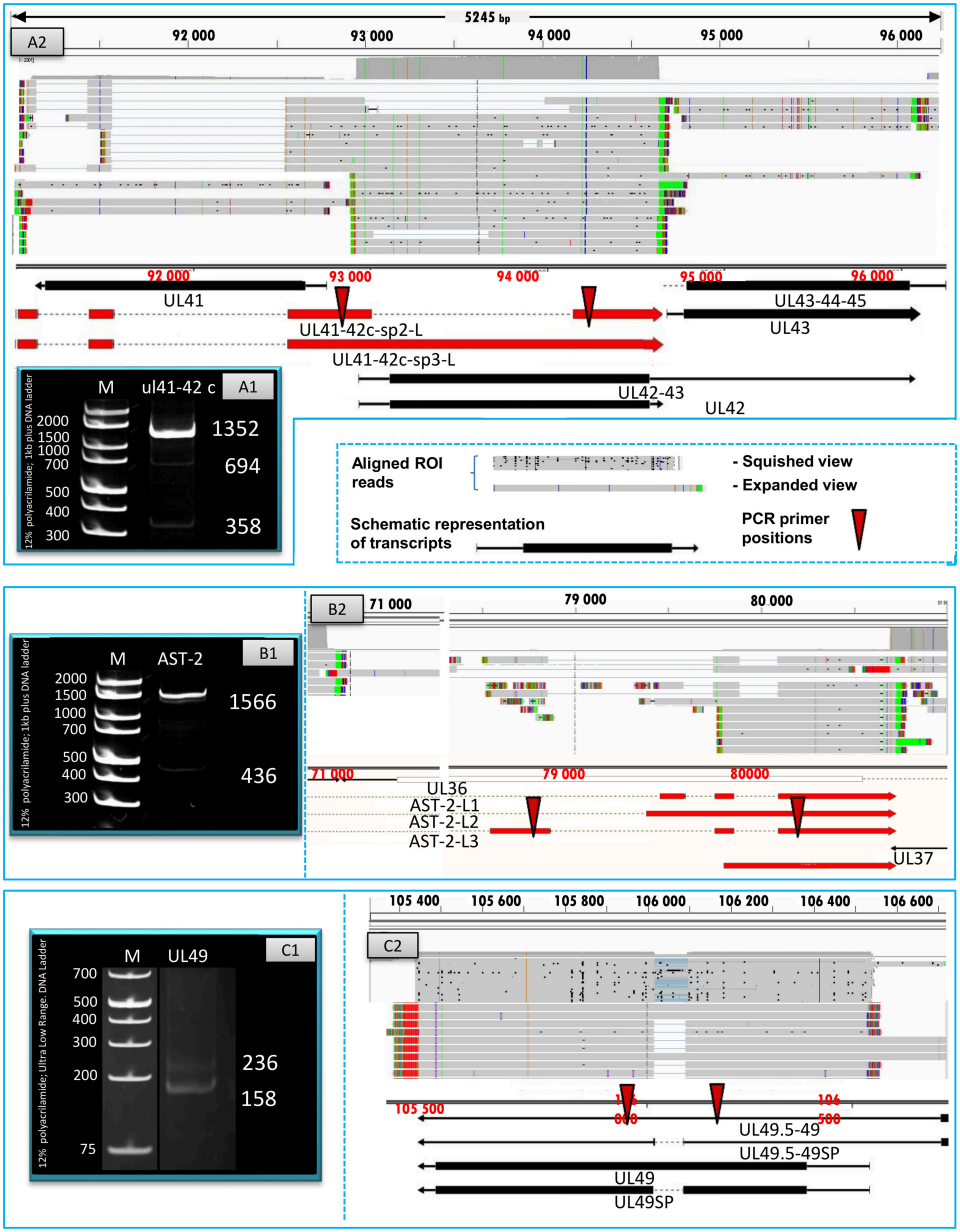
Splice isoforms. PCR validation of the splice sites of the UL41-42 complex transcript **(A1)**, AST-2 transcript **(B1)**, and the UL49 RNA **(C1)**. **(A2)** Shows the coverage of reads across the examined genomic region, the ROIs (gray rectangles) and the schematic representation of the genes (red or black arrows). **(B2)** Illustrates the expanded view of ROIs, the read coverage along analyzed region and the schematic view of AST-2 and adjacent genes. **(C2)** Graph shows the read coverage, the ROIs and the schematic representation of the examined region. The numbers on the left side of panels **A1, B1**, and **C1** indicate the sizes of DNA ladders while the numbers on the right side of these panels indicate the sizes of the PCR products amplified from the splice junction regions of the various splice isoforms (see Table 5). **(A1)** The 1,358-bp band shows the unspliced, while the 694- and the 388-bp bands show the two splice variants of the UL41-42 transcripts. **(B1)** The 1,566-bp indicates the unspliced, while the 436-bp band show the spliced version of AST-2 transcript. **(C1)** The 236-bp band shows the unspliced, while the 158-bp band shows the spliced isoform of UL49 mRNA. The colors indicate the poly(A) tails of the transcripts; green: from left to right orientation, red: from right to left orientation. The striped colors indicate the Clontech adaptor (universal primer binding site) used for the PCR amplification. They are labeled as such because they do not map to the PRV genome. The vertical red arrows indicate the precise location of the forward and reverse PCR primers on the HSV genome.

In this report, we detected 13 novel spliced transcripts (Figures 1, 3, Table 5). The most intricate splicing pattern was found in the *ul41-42* and *ul41-45* complex transcripts (Figure 3). We identified two splice variants of the AST-2 antisense transcript. We also detected a new splice isoform of the *ul49* gene, which was found to be expressed in a much higher level than the non-spliced variant. The UL49 and UL49-48 transcripts are the only ones in which the splicing occurs within an ORF, which is translated (in other cases the splicing takes place in either non-coding transcripts or in the downstream genes in polycistronic transcripts, which are non-translated). The splicing within the *ul49* ORF results an in-frame deletion.

**Table 5:** Novel splice isoforms.

**Spliced transcripts**	**TSS**	**TES**	**Exon 1**	**Exon 2**	**Exon 3**	**Exon 4**
AST-2-L1	79,449	80,425	79,944–79,581	79,743–79,883	80,091–80,725	
AST-2-L3	78,531	80,425	78,531–78,852	79,743–79,883	80,091–80,725	
UL41-42 c sp-1	91,067	94,656	91,067–91,122	91,414–91,552	92,536–92,982	94,006–94,656
UL41-42 c sp-2	91,067	94,656	91,067–91,122	91,414–91,552	92,536–92,982	94,148–94,656
UL41-42 c sp-3	91,067	94,656	91,067–91,122	91,414–91,552	92,536–94,656	
UL41-42 c sp-3-L	91,013	94,657	91,013–91,122	91,414–91,552	92,536–94,656	
UL41-42 c sp-4	91,526	94,656	91,526–91,552	92,536–946,56		
UL41-42 c sp-4-L1	91,457	94,656	91,457–91,552	92,536–94,656		
UL41-42 c sp-4-L2	91,299	94,656	91,299–91,552	92,536–94,656		
UL41-45c sp	91,067	98,691	91,067–91,122	97,948–98,691		
UL49-48 sp	106,538	103,515	106,091–106,538	103,515–106,017		
UL49 sp	106,543	105,442	106,091–106,543	105,442–106,017		
UL49.5-49 sp	106,718	105,442	106,091–107,128	105,442–106,017		

*PacBio sequencing revealed a new splice isoform transcribed from the ul49 gene (we were able detect the same intron within three transcripts: UL49; UL49-48; UL49.5-49). We also identified seven additional spliced transcripts in two genomic regions: two different splice variants of the AST-2 transcript, four splice variants of the UL41-42 complex transcript, as well as a spliced version of the UL41-45 complex RNA. This table shows the novel spliced transcripts, as well as their TSS and PAS positions and the coordinates of the exon boundaries. The same colors indicate the same positions in the viral genome*.

### Novel polycistronic transcripts

According to the current concept, the HSV genome is organized in such a way that the downstream genes in a tandem gene cluster are transcribed either as monocistronic transcripts or as downstream genes of polycistronic (bi-, tri-, tetra-, or penta-cistronic) RNA molecules, whereas the upstream genes are expressed exclusively as parts of polycistronic transcripts. Our earlier investigations revealed that several upstream genes of 3′-coterminal gene clusters of the PRV are also transcribed as monocistronic RNA molecules (Tombácz et al., 2016). However, in this study, with the exception of the transcripts expressed from the embedded genes, we only detected novel polycistronic molecules (Table 6) that include transcripts terminated upstream of the co-terminal PA sites. Most of the newly discovered polycistronic RNA molecules are expressed at low levels, which explain why they had previously gone undetected.

**Table 6:** Novel polycistronic and complex transcripts.

**Transcripts**	**TSS**	**TES**
UL2.5-3-L	10,323	11,735
UL5.5-4	13,518	11,732
UL6.5-7	16,433	18,060
UL12.7-11	25,781	24,782
UL22-23	43,818	47,906
UL34.5-35	69,732	70,957
UL39.5-40	88,773	91,001
UL39.2-40	89,001	91,001
UL42-43	92,936	96,081
UL44.5-45	96,620	98,691
UL53.5-54	112,508	115,302
US8-8.5	141,170	143,275
US8.7-9	142,071	143,687
UL43-44-45	94,653?	98,691
UL52-53-53.5	108,985	113,461
US7-8-8.5-9	139,698	143,687
US3-4-5-6-7	135,187?	141,034
UL16-17-15 c	33,204?	34,813
UL23-24 c	49,538?	46,607
UL41-42 c	91,702?	94,655
UL41-42 c sp-1	91,067	94,656
UL41-42 c sp-2	91,067	94,656
UL41-42 c sp-3	91,067	94,656
UL41-42 c sp-4	91,523	94,656
UL41-45 c sp	91,067	98,691
UL49.5-50 c	106,120?	108,173
UL52-51 c	112,131	108,265

*This table shows the newly discovered polycistronic and complex transcript isoforms. The “?” indicates that we were unable to determine the exact 5′ end of the given transcripts*.

### Complex transcripts

Complex transcripts are defined as containing at least two genes with opposite orientations. We identified 10 full-length complex transcripts in HSV, seven of which had been generated by alternative splicing of the RNA molecule encoded by the genes within the *ul41*-*45* region (Figure 1, Table 6). Earlier we had detected the homologs of the UL41/42 and the UL52/51 complex transcripts of HSV-1 also in PRV (Tombácz et al., 2016). Our investigations revealed a widespread expression of very long complex transcripts in PRV whose upstream sequences could not be determined even with the long-read PA-Seq technique. We detected more full-length but fewer partial complex transcripts in HSV than in PRV; the reason for the latter may be that in our previous report we also used random primer-based sequencing that allowed for the capture of more distal upstream sequences than the PA-Seq technique alone. The RNA molecules with partial sequences were illustrated as if they were controlled by the promoters of the closest upstream genes and were given *ad hoc* names accordingly. However, it is possible that they are shorter and initiated by yet unidentified promoters, or even longer and driven by more distal cis-regulatory sequences.

### Transcriptional overlaps between HSV genes

In this part of the study, we investigated the transcriptional overlaps along the entire HSV genome.

#### Parallel overlaps

**Figure 4:**
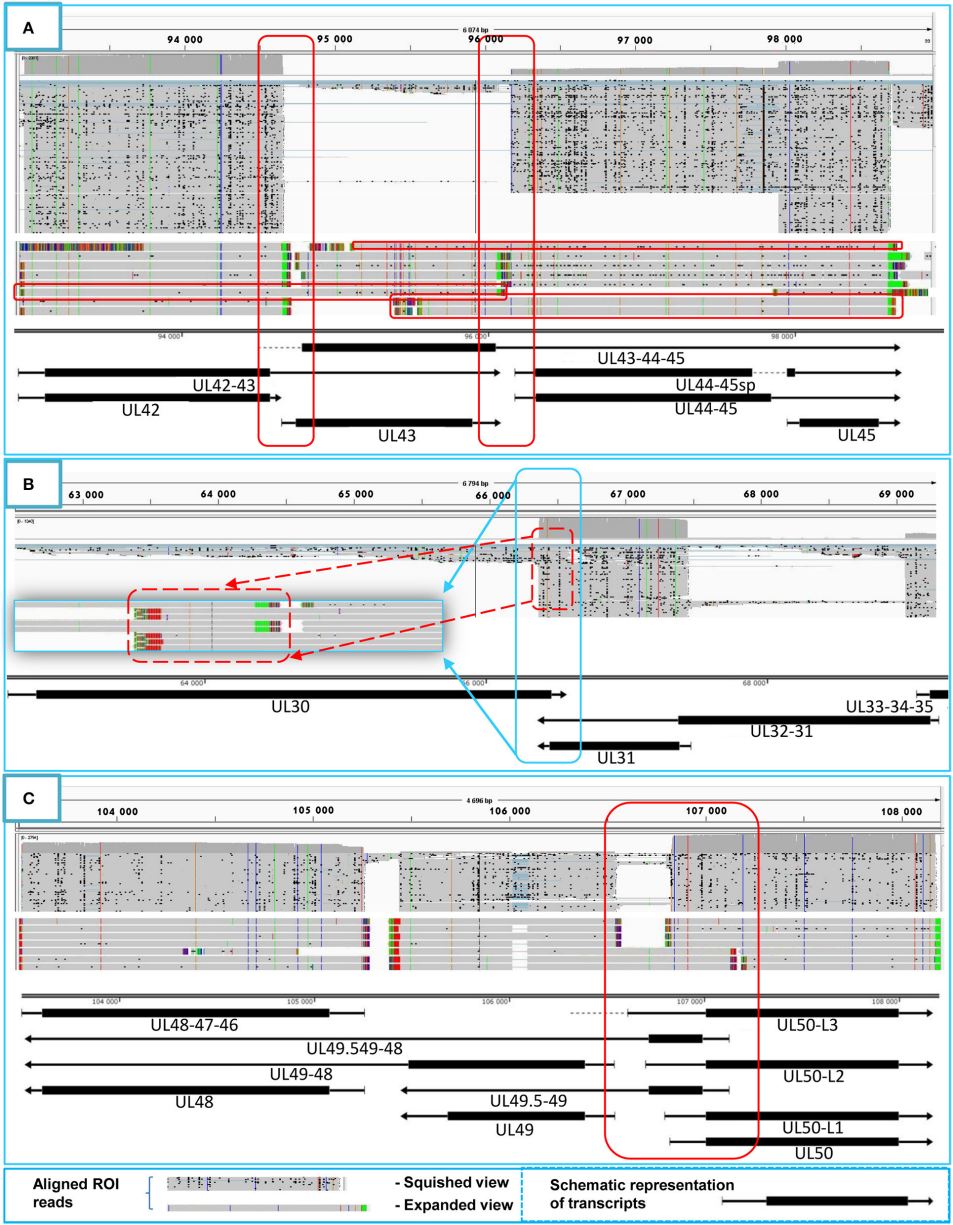
Transcriptional overlaps. Transcriptional overlaps between adjacent genes. **(A)** Represents the parallel transcriptional overlap exemplified by the *ul42, ul43*, and *ul44* genes, which is a unique genomic region of HSV-1 in the sense that the transcripts from these genes have their own transcription termination sites and only rarely form polycistronic transcripts. **(B)** Illustrates convergent “hard” transcriptional overlaps using the *ul30* and *ul31* genes as an example. The TES sequences of these genes are located within the transcribed region of the partner gene. **(C)** Illustrates the divergent transcriptional overlaps with the example of *ul49.5* and *ul50* genes. It can be seen that all of the isoforms of the UL50 transcript exhibit a large extent of overlap with the UL49.5 RNA molecules. The panels of this figure show the ROIs (gray color; squished and expanded view) and the coverage of reads across the given genomic regions. The overlapping transcripts are shown at the bottom of the figures. The colors indicate the poly(A) tails of the transcripts: green: from left to right orientation, red: from right to left orientation. The striped colors indicate the Clontech adaptor used for the PCR amplification.

Tandemly-oriented adjacent HSV genes can either partially or fully overlap one another. We detected 115 novel parallel full transcript overlaps, including the 34 5′ truncated genes, four 3′ truncated genes and 18 adjacent genes, as well as six partial tail-to-head overlaps between tandem genes. This latter kind of overlap is illustrated in Figure 4A, using *ul42, ul43*, and *ul44* genes as examples. As a result of this analysis, we obtained that HSV contains fewer ORF-overlaps between the adjacent tandem genes than does PRV (7 vs. 8). However, there are many more embedded genes were detected in HSV.

#### Convergent overlaps

The tail-to-tail overlaps can be “hard” when all of the adjacent transcripts overlap each other or “soft” when both overlapping and non-overlapping transcripts are produced from the same gene. We detected the UL30/31-32, UL1-2-3/4-5, AST-1/UL37, and the AST-2/UL37 transcripts, which represent the only “hard” overlaps in HSV. The UL30/31-32 overlap is 46 nts longer than the earlier annotation suggests (McGeoch et al., 1988), and it is also present in PRV (Tombácz et al., 2016) (Figure 4B). Furthermore, very short (3–10 nts) intergenic regions were detected between the convergently-oriented gene pairs *ul4*/*ul3.5, ul35-36, ul45-46, AST-3-ul48, AST-1-ul29*, which potentially lead to interference between the RNA polymerase molecules when these simultaneously synthesize mRNAs on the neighboring genes, since transcription normally does not stop at the TES.

#### Divergent overlaps

Earlier annotations revealed that three out of 13 divergently-oriented HSV gene pairs form head-to-head overlaps with each other (Figure 4C), including 5′-UTR/5′-UTR, the 5′-UTR/ORF, and ORF/ORF overlaps. PacBio analysis revealed alternative TSSs for several genes, which generate overlaps or much longer overlapping regions than published earlier (12 out of 13).

### Detection of antisense RNAs with sequence-specific qRT-PCR

**Figure 5:**
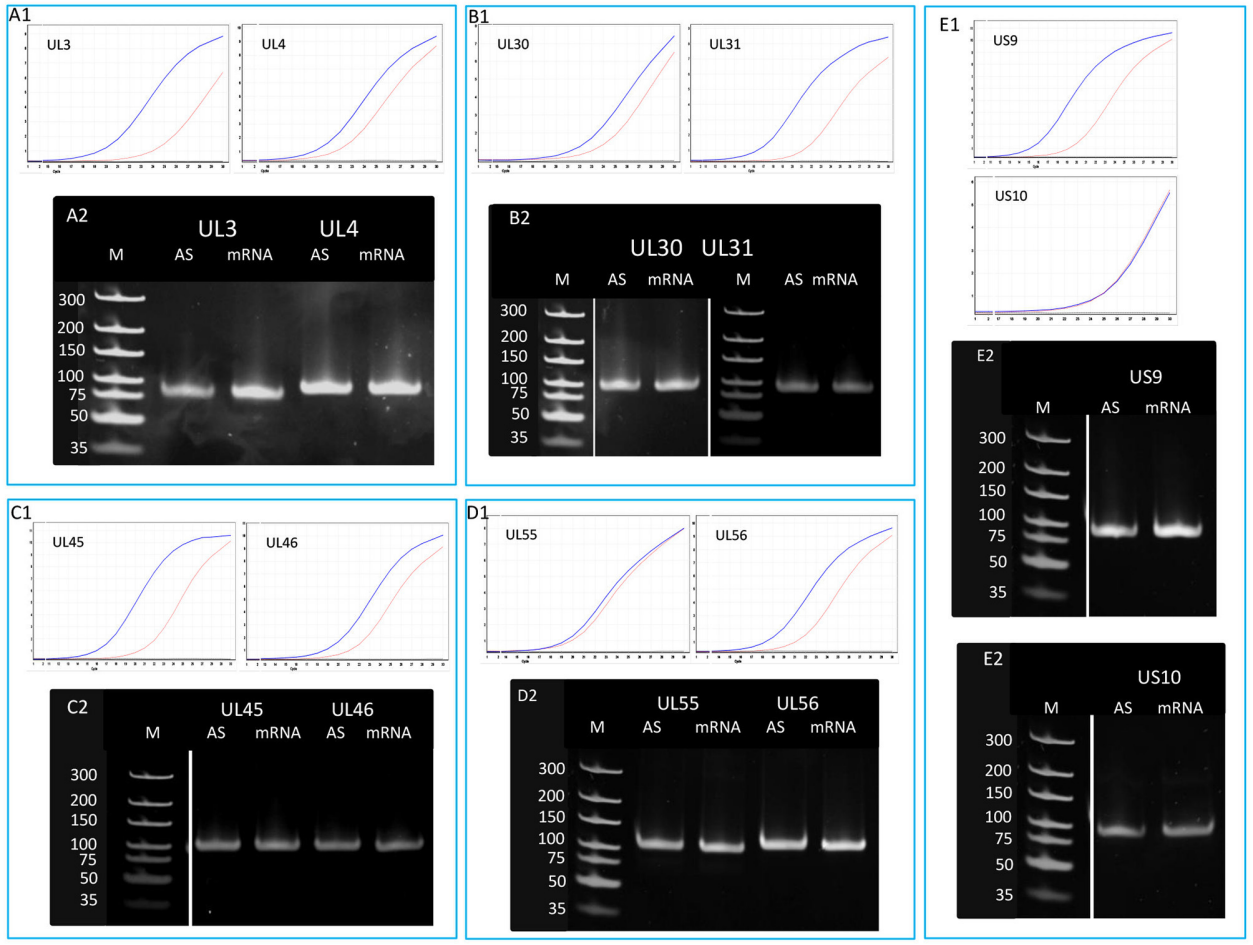
Detection of the antisense RNAs produced from the complementary DNA strands of 10 convergent HSV genes. A quantitative RT-PCR technique was used for the analysis of potential asRNAs derived from the transcriptional read-through between convergent genes. The amplification curves obtained by qRT-PCR are shown to demonstrate the specificity of the amplification. The curves of specific transcripts cross the threshold line within 18.3–22.5 cycles, while the curves of NO-RT controls remain flat. Panel **(A1)** shows the expression curves of the transcripts produced from plus and minus strands of the *ul3* and *ul4* convergent gene pair. Panel **(A2)** shows PCR products of the UL3 and UL4 transcripts and their antisense partners. Panel **(B)** shows the qRT-PCR amplification curves **(B1)** and the PCR products **(B2)** of UL30 and UL31 mRNAs and their asRNA partners. **(C1)** qRT-PCR curves of UL45 and UL46 mRNAs and the asRNAs produced from the complementary strands of these genes. **(C2)** PCR amplification products from the *ul45*-*ul46* genomic region. **(D1)** Amplification curves of the UL55 transcript and its convergent partner UL56, as well as the antisense transcripts produced from the complementary DNA strands. **(D2)** Gel electrophoresis of the PCR products amplified from cDNAs UL55 and UL56 HSV mRNAs and their antisense partners. Panel **(E)** illustrates the qRT-PCR expression curves **(E1)** and the PCR products **(E2)** of US9-US10 mRNAs and their asRNAs. Gel samples were collected after saturation.

In order to determine the existence of additional asRNAs, which are undetected by sequencing due to the potential lack of PA tails or being too long to be sequenced by the PacBio technique, five convergent HSV gene pairs were selected for the analysis of transcriptional activity of the complementary DNA strand by qRT-PCR. The asRNAs are supposed to be produced by transcriptional read-through from the convergent partner genes. We detected varying levels of asRNAs from each examined locus (Figure 5). The standard deviation (SD) values of the three parallel experiments are presented in Table S5. The pairwise comparison of qPCR curves shows that the relative amounts of asRNAs in 8 out of 10 genes are much lower than those of their mRNA partners, except in *ul54* gene where the mRNA level only slightly exceeds that of the asRNA, and in the *us10* gene where the sense-antisense partners are expressed at a similar rate. The reason for this latter phenomenon could be that the *us10* gene is expressed in low level, and therefore even a weak antisense RNA expression can produce a comparable level of transcripts.

### The expression of convergent genes exhibits an inverse pattern

**Figure 6:**
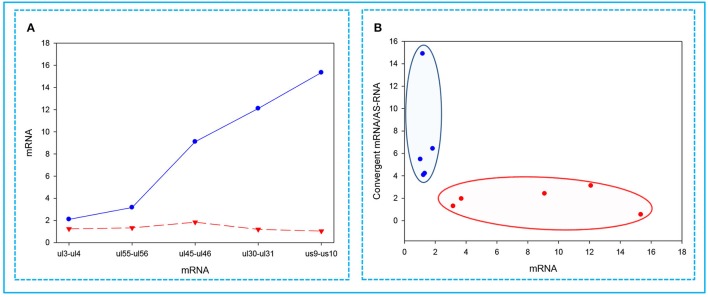
Correlations between the expressions of convergent HSV genes. The relative copy numbers (R_*x*_ values) were calculated for the transcripts encoded by five convergent gene pairs: *ul3*-*ul4*; *ul30*-*ul31; ul45*-*ul46; ul55*-*ul56; us9*-*us10*. **(A)** The less abundant mRNAs encoded by one of the convergent gene pairs are indicated by a red dashed line, while the more abundant partner mRNAs are depicted by a blue solid line. The figure shows that the higher the relative abundance of a transcript in the total PRV transcriptome, the lower the level of mRNA produced from the convergent gene. **(B)** Cluster analysis of the R_x_ values of the examined mRNAs compared to the ratio between the mRNA/antisense transcripts produced by the convergent gene partner. This figure shows that if a high mRNA levels are produced from a gene, then it exerts a decrease of mRNA/asRNA ratio for the convergent gene. The reason for this is that asRNAs are supposed to be produced as a result of transcriptional read-throughs from the oppositely-oriented genes. The blue dots illustrate low-abundance, while the red dots represent the high-abundance mRNAs.

In this part of the study, we observed the phenomenon that high transcriptional activity of a gene is associated with a low level of mRNAs from the convergent genes. We found that the higher the expression of a gene, the lower the expression of the convergent partner was (Figure 6A). Furthermore, we also observed that a gene with a high expression rate has a convergent partner with a low mRNA/asRNA ratio, and conversely, the partners of genes with low expression rates have higher values (Figure 6B).

### Read count normalization by spike-in DNA

**Figure 7:**
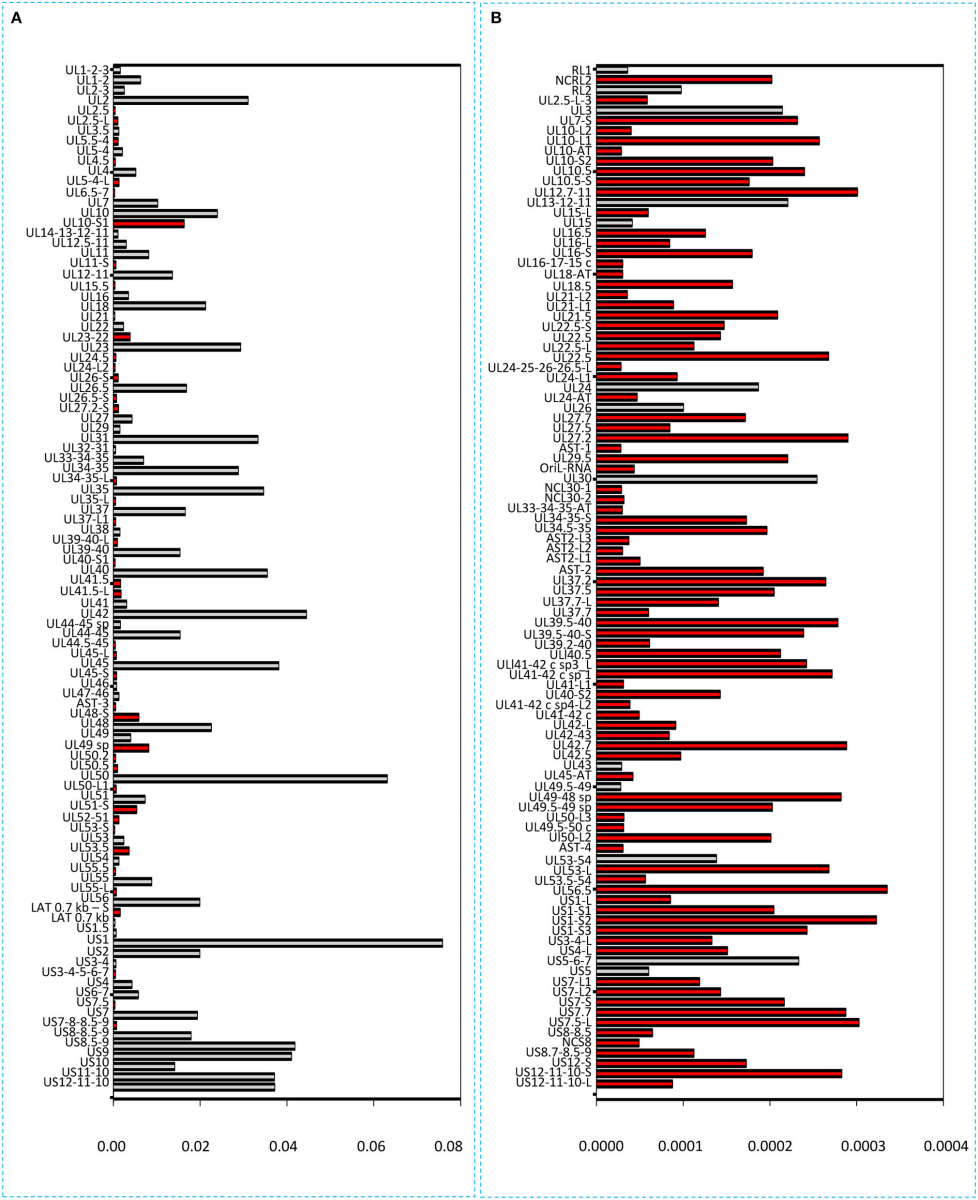
Bar chart representation of the HSV read counts using the marker-normalized values. **(A)** Horizontal bars represent the normalized read counts of the most abundant 100 HSV-1 transcripts. The novel transcripts are labeled by red. **(B)** This chart shows the less abundant 100 transcripts (101–200 in rank) of HSV. Red color indicates the newly identified transcripts. The scale (x-axis) of the two parts of the figure is different (200-fold difference). The transcripts are ranked on the basis of their genomic positions.

**Figure 8:**
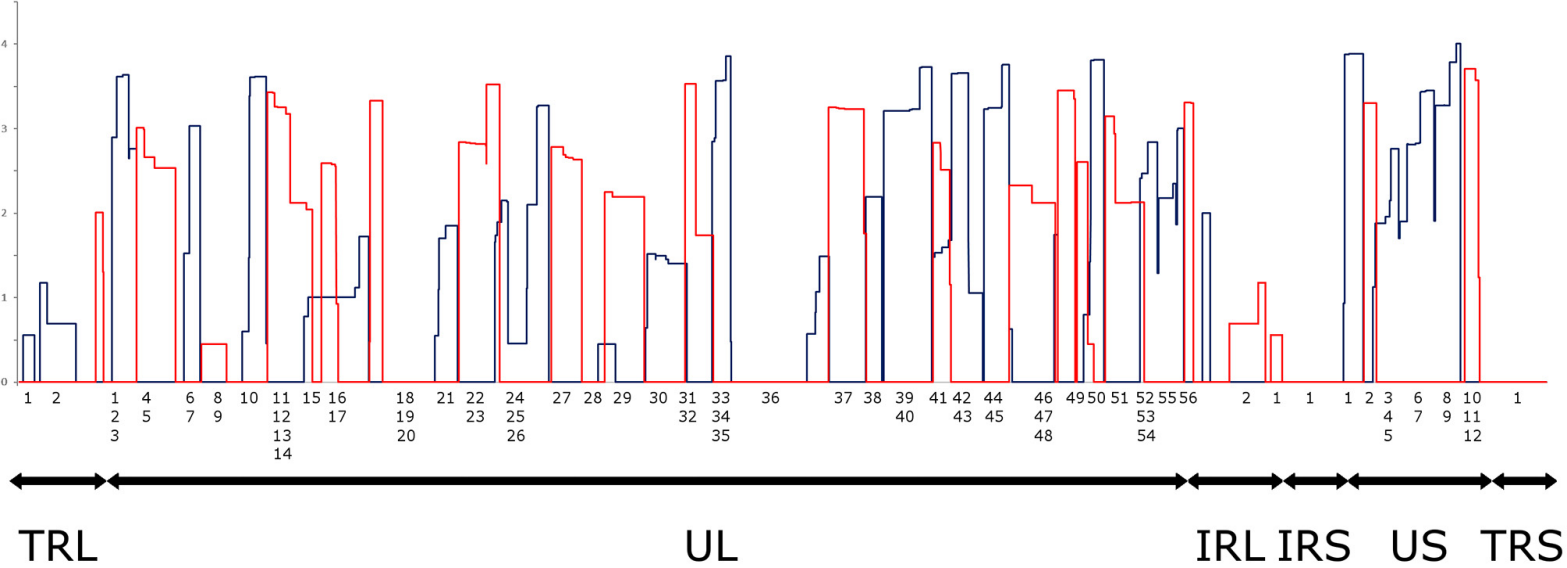
Transcriptional activity throughout the HSV genome. Transcriptional activity was calculated by normalizing the read counts to the read length distributions determined by equimolar molecular-weight size markers. The expression values of overlapping transcripts were summed for each nucleotide. The blue histogram shows the expression values on the plus strand, while the red histogram shows the expression values on the minus strand. All values are shown on a logarithmic scale. The genomic regions are marked under the histogram (TRL, Long Terminal Repeat; UL, Unique Long; IRL, Long Internal Repeat; IRS, Short Internal Repeat; US, Unique Short; TRS, Short Terminal Repeat). Note that an RNA population from several different time points p.i. was sequenced, where late genes are presumably overrepresented.

It has been reported in our recent publication (Tombácz et al., 2017) and by others (O'Grady et al., 2016) that library preparation methods for PacBio sequencing enrich and sequence 1–2 kb-long fragments. Consequently, the long (>3 kb) or the short (<700 nts) transcript isoforms are underrepresented in the raw number of reads. To account for this bias, raw read counts were normalized by spike-in DNA (Figure 7). This analysis represents global gene expression along the HSV genome in a sample containing a mixture of RNA molecules collected from six different time points of viral infection. We also calculated the transcriptional activity throughout the HSV genome using the same method (Figure 8).

## Discussion

Short-read sequencing has become a common technique for the annotation of transcriptome datasets (Mortazavi et al., 2008; Wang et al., 2009; Djebali et al., 2012). However, these methods are not optimal for resolving transcript structures, especially for the *de novo* characterization of complex transcript isoforms and long overlapping RNAs. In this study, a PCR-based long-read sequencing method was applied for the analysis of the HSV-1 transcriptome. Our investigations revealed a much higher level of transcriptomic complexity than has been captured by existing annotations. Here we provide a base-pair-precision annotation of already detected but not fully characterized RNA molecules. We have also succeeded in identifying 34 previously undescribed putative protein-coding transcripts, which were all transcribed from the ORF region of already annotated HSV-1 genes. We were able to identify in-frame AUG triplets for all genes in relative close position to the TSSs. Ten putative ncRNAs have also been detected. Four of these were 3′ truncated RNA molecules expressed from the promoter of protein-coding HSV-1 genes, but they were prematurely terminated from an internal PA signal. These transcripts lack stop-codons, and therefore we assume that they are lncRNAs. The homolog of HSV-1 NCS8 transcript has also been described in PRV in a recent report (Tombácz et al., 2016). This study identified many polycistronic and complex transcripts. Two complex transcripts of HSV show homology to previously described PRV transcripts (Tombácz et al., 2016), while the rest of them appear to be unique to HSV. We have also detected pervasive asRNA expression throughout the entire HSV genome with both cDNA sequencing and PCR. The antisense RNAs either have their own promoters, or they are transcribed from the promoter of neighbor or of distal genes as being transcriptional read-through products. The question as to whether the long transcripts are the result of transcriptional noise, if they contribute to the viral proteome, or if they fulfill other roles, remains to be ascertained. Another possibility is that these long transcripts are mere byproducts of a gene regulation mechanism that is based on interference between the transcriptionally active genes (Boldogköi, 2012).

No short ncRNAs homologous to CTO-S or PTO of PRV located near the replication origins were detected in HSV. However, we identified Ori-overlapping HSV transcripts, which were the long TSS variants of genes *us1* and *us12*, as well as a putative short ncRNA overlapping Ori-L. The existence of these transcripts may indicate a crosstalk between the replication and transcription machinery.

Rutkowski and colleagues have recently investigated the translation of HSV-1 transcripts throughout the viral life cycle with a ribosome profiling technique using the RPKM (reads per kilobase per million mapped reads) model (Rutkowski et al., 2015). However, they mapped only the known HSV-1 transcripts, and provided no information from the translational start sites, and therefore we cannot check whether our novel transcripts (e.g., the 5′ truncated or the antisense RNAs) are translated or not.

PacBio long-read sequencing revealed the existence of many transcript isoforms including TSS, TES, and splice variants produced from the same gene. Additionally, as it has been detected in PRV (Tombácz et al., 2016), HSV transcripts also exhibit minor variations in both of their 5′ and 3′ termini.

Our investigations revealed an intricate meshwork of transcriptional read-throughs leading to overlapping RNA molecules. It turned out that HSV genes are transcribed in more combinations than it had been previously thought. The number of asRNAs and the complex transcripts of HSV are likely to be underestimated, because most of them may have been undetected due to their non-polyadenylated nature or because they are too long to be identifiable with even a long-read platform. This hypothesis was tested by examining the transcriptional activity of 10 HSV genes by qRT-PCR, which detected asRNAs in each case, suggesting that the entire HSV genome expresses long overlapping transcripts.

The analysis of antisense transcripts and their mRNA partners has disclosed the interconnected regulation of transcription (Katayama et al., 2005), and in many cases reciprocal patterns of gene expression (Lehner et al., 2002). Additionally, asRNAs have been shown to inhibit the transcription of complementary mRNA in human cells (Izant and Weintraub, 1985). In this work, we show that high mRNA expression from a gene was associated with low mRNA levels produced from the convergent partner. Additionally, high expression rates from genes were also associated with a low mRNA per asRNA ratio for convergent genes. We explain this phenomenon by transcriptional read-throughs, which are believed to result in the collision of the transcriptional machineries. The extensive transcriptional read-throughs and inverse correlations between the expression of convergent genes provide further support to our TIN hypothesis, which claims that genes interact with each other at the level of transcription throughout the entire genome (Boldogköi, 2012).

Finally, in this study, we developed a method for the quantification of RNA molecules based on calculations using ROI reads, which differs from the approach that we published earlier (Tombácz et al., 2017) in two important aspects: first, instead of the non-amplified SMRT method, here we used an amplified Iso-Seq technique, and second, we also sequenced spike-in DNAs for normalizing the size-dependent loading of cDNA molecules into ZMWs of the PacBio SMRT-cells, which allowed for comparison of the relative amounts of transcripts of different lengths.

In summary, our investigations essentially redefine the transcriptome of HSV-1. We demonstrated that HSV-1 exhibits a far higher transcriptional complexity than predicted from *in silico* ORF-based genome annotations or than those detected by techniques that map the termini of the transcripts. Most of the novel RNA molecules are produced at low levels which hinders their detection by gel-based assays or, because of their overlapping nature, even by PCR. Additionally, our analysis significantly increased the number of already known transcriptional read-throughs between the genes.

## Accession number

Raw sequencing files, processed data files as well as metadata have been submitted to the NCBI GEO repository and can be found with GenBank accession number GSE97785.

## Author contributions

ZBo, DT, and MS conceived and designed the study. DT, ZC, DS, and ZBo performed the experiments. DT, ZBo, AS, NM, ZBa, and DS analyzed the data. ZBo and DT wrote the manuscript. ZBo coordinated the project. All authors took part in the revision, read and approved the final manuscript. Funding acquisition: MS, ZBo, DT.

### Conflict of interest statement

The authors declare that the research was conducted in the absence of any commercial or financial relationships that could be construed as a potential conflict of interest.

## Supplementary material

Table S1 The list of the primers used in this study.

Table S2 The list of the predicted promoter elements.

Table S3 The list of the predicted poly(A) signals.

Table S4 The of the identified HSV-1 genes.

Table S5 The SD values of the parallel experiments used in the qRT-PCR analysis.
