# Supplementary material for: Long-Read Isoform Sequencing Reveals a Hidden Complexity of the Transcriptional Landscape of Herpes Simplex Virus Type 1
Source: Front Microbiol. 2017 Jun 20;8:1079. doi: 10.3389/fmicb.2017.01079 (PMC5476775; doi:10.3389/fmicb.2017.01079)
Supplement: Table S5 — The SD values of the parallel experiments used in the qRT-PCR analysis. [file Table5.DOCX]

| ***Gene*** | | **Ct** | | **Efficiency** | |  | ***Gene*** | | **Ct** | | **Efficiency** | |
| --- | --- | --- | --- | --- | --- | --- | --- | --- | --- | --- | --- | --- |
|  |  | **Mean** | **SE** | **Mean** | **SE** |  |  |  | **Mean** | **SE** | **Mean** | **SE** |
| ***ul3*** | ***mRNA*** | 17,10 | 0,20 | 1,61 | 0,01 |  | ***ul4*** | ***mRNA*** | 17,10 | 0,00 | 1,71 | 0,00 |
|  | ***AS*** | 20,77 | 0,30 | 1,61 | 0,01 |  |  | ***AS*** | 18,73 | 0,04 | 1,68 | 0,01 |
|  | | | | | |  |  | | | | | |
| ***ul30*** | ***mRNA*** | 18,60 | 0,07 | 1,63 | 0,01 |  | ***ul31*** | ***mRNA*** | 13,70 | 0,13 | 1,63 | 0,01 |
|  | ***AS*** | 20,73 | 0,10 | 1,63 | 0,00 |  |  | ***AS*** | 17,50 | 0,09 | 1,73 | 0,03 |
|  | | | | | |  |  | | | | | |
| ***ul45*** | ***mRNA*** | 13,83 | 0,15 | 1,66 | 0,01 |  | ***ul46*** | ***mRNA*** | 16,77 | 0,20 | 1,67 | 0,01 |
|  | ***AS*** | 17,23 | 0,23 | 1,67 | 0,01 |  |  | ***AS*** | 18,33 | 0,08 | 1,68 | 0,01 |
|  | | | | | |  |  | | | | | |
| ***ul55*** | ***mRNA*** | 16,43 | 0,16 | 1,72 | 0,00 |  | ***ul56*** | ***mRNA*** | 15,53 | 0,07 | 1,68 | 0,00 |
|  | ***AS*** | 17,07 | 0,11 | 1,71 | 0,01 |  |  | ***AS*** | 18,03 | 0,05 | 1,66 | 0,01 |
|  | | | | | |  |  | | | | | |
| ***us9*** | ***mRNA*** | 12,23 | 0,08 | 1,70 | 0,01 |  | ***us10*** | ***mRNA*** | 18,33 | 1,00 | 1,67 | 0,03 |
|  | ***AS*** | 15,90 | 0,12 | 1,67 | 0,00 |  |  | ***AS*** | 20,07 | 0,44 | 1,64 | 0,00 |
